# Investigating Setup Error Differences between Conventional and Ultra‐Hypofractionated Whole Pelvis Radiation Therapy: A Secondary Analysis from the HOPE Randomized Clinical Trial

**DOI:** 10.1002/acm2.70646

**Published:** 2026-05-31

**Authors:** Bryan Schaly, Lucas C. Mendez, Maha Khawaja, Alec Black, Andrew Warner, Douglas A. Hoover

**Affiliations:** ^1^ Department of Radiation Oncology London Health Sciences Centre London Ontario Canada; ^2^ Department of Medical Biophysics Western University London Ontario Canada; ^3^ Department of Health, Aging and Society McMaster University Hamilton Ontario Canada

**Keywords:** fiducial markers, planning target volume margins, prostate cancer, setup errors, ultra‐hypofractionation

## Abstract

**Introduction:**

The multi‐institutional HOPE trial (NCT04197141) compared conventional (25 fractions over 5 weeks) to ultra‐hypofractionated (5 fractions over 1.5 weeks) whole pelvis radiotherapy for unfavorable intermediate and high/very high‐risk prostate cancer patients treated with brachytherapy boost. Within this randomized controlled trial, we investigated setup errors for clinical target volumes corresponding to the prostate (CTVp) and nodes (CTVn).

**Methods:**

Fifty‐four patients treated within a single institution were analyzed, with 28 treated on the ultra‐hypofractionated arm and 26 on the conventional fractionation arm. Prior to treatment, three fiducial markers were implanted into the prostate for daily cone‐beam computed tomography (CBCT) matching. All plans were delivered using volumetric modulated arc therapy with a uniform 6‐mm PTV margin. For all treatment fractions, average fiducial matching errors were measured for CTVp, while errors to CTVn were determined based on the difference between the treatment position and an automatic match to bone. Asymmetric PTV margins to account for prostatic and nodal setup errors were calculated using the van Herk formalism.

**Results:**

Median (interquartile range [IQR]) 3D errors for CTVp were 1.4 (0.8–2.5) mm for conventional versus 1.2 (0.7–1.9) mm for the ultra‐hypofractionatated arms (*p* = 0.013), while for CTVn they were 2.8 (1.6–4.2) mm and 2.9 (2.2–4.4) mm, respectively (*p* = 0.07). Between arms, statistically significant differences in setup errors were noted in all directions for CTVp and in the anterior‐posterior direction for CTVn (*p* < 0.05). To account for setup error, the required PTV margins in the anterior‐posterior/superior‐inferior/lateral directions were 2.8/3.8/0.8 and 2.7/3.0/1.0 mm for conventional and ultra‐hypofractionated treatments, respectively; for elective nodes, margins were 5.4/4.4/0.7 and 5.7/4.9/1.0 mm, respectively.

**Conclusions:**

The 6‐mm PTV margin around the CTVn is appropriate to account for residual setup error while the margin around the CTVp could be reduced, assuming other sources of error are controlled. The residual 3D errors for the prostate were statistically significantly smaller in the ultra‐fractionated regimen, which was potentially due to longer image matching times and/or the higher percentage of patients treated with a 6‐DOF couch in that arm. However, this came at the expense of slightly larger nodal PTV margins in the ultra‐hypofractionated arm. These findings highlight the need for individual centers to independently quantify their treatment uncertainties, as measurable differences were observed between treatment arms despite identical image guidance protocols.

## 1 INTRODUCTION

In the radiation treatment of prostate cancer, hypofractionated radiotherapy has become the preferred treatment approach due in part to the radiobiologic properties of prostate cancer tumors.^1^ Clinical evidence has shown that treatment of the prostate and regional pelvic lymph nodes is well tolerated in 20 fractions, and this regimen has become an accepted new standard‐of‐care option for external beam radiotherapy (EBRT).^2^ However, when combined with brachytherapy, EBRT to the prostate and pelvic nodes is typically delivered using 1.8–2 Gy per fraction. More recently, the phase 2 multicenter clinical trial, HOPE, compared the standard EBRT regimen of 45 Gy in 25 fractions against 25 Gy in 5 fractions to the prostate and pelvic lymph nodes, following high dose rate brachytherapy (HDR) boost of 15 Gy in 1 fraction. In this trial, no significant differences in acute toxicity or patient reported outcomes were noted between arms.^3^ Likewise, the ultra‐hypofractionated arm was found to be non‐inferior to the standard arm with respect to the late gastrointestinal (GI) and genitourinary (GU) toxicities. The HOPE trial used a uniform planning target volume (PTV) margin of 6 mm surrounding the prostate and pelvic lymph nodes in both arms. Given the positive results from this trial, ultra‐hypofractionation may become a standard‐of‐care option, and so it is imperative to examine the required prostate and nodal PTV margins to reduce unnecessary toxicity. Although there are studies of interfraction prostate motion for conventional fractionation^4^, ^5^, ^6^, ^7^ and hypofractionation,^8^ similar studies for ultra‐hypofractionated radiation therapy (≤5 fraction) of prostate and regional lymph nodes are scarce. Kishan et al.^9^ determined that matching fiducial markers in stereotactic radiation therapy of the prostate while simultaneously treating the pelvic lymph nodes can lead to underdosing the nodal PTV, mainly due to large differences in marker shift with respect to the bony anatomy. Tyagi et al.^10^ determined that a 5 mm margin around the pelvic lymph nodes provided adequate coverage in ∼75% of their patient cohort. Wu et al.^11^ determined that asymmetric PTV margins of up to 8–9 mm were required for simultaneous boosting of gross nodal targets when matching prostate fiducial markers. The goal of our study is to determine the required PTV margins to safely deliver this type of treatment following the image guidance protocol within the HOPE trial, which included daily cone‐beam computed tomography (CBCT) imaging with fiducial markers implanted into the prostate. However, the image matching process allowed shifting off the fiducial markers to ensure pelvic node coverage, thereby resulting in a “best‐fit” match of the prostate and pelvic nodes.

## 2 METHODS

### 2.1 Patient data

Eighty patients were enrolled in the HOPE trial of which 54 patients were treated at our institution and included in this secondary analysis under an institutionally approved protocol (Western University REB #114388). Twenty‐eight of these patients were treated on the experimental arm (5 fractions) while 26 patients were treated on the standard‐of‐care arm (25 fractions). All patients received 15 Gy in one fraction of HDR brachytherapy followed approximately 2 weeks later by EBRT. Three fiducial markers were inserted into the prostate during the brachytherapy procedure to aid in prostate localization during EBRT. In all treatments, the prostate and at least 1 cm of the proximal seminal vesicles were included in the prostate clinical target volume (CTVp), while obturator, pre‐sacral, external and internal iliac lymph nodes were included in the nodal CTV (CTVn). The planning target volume (PTV) used a 6 mm uniform margin expansion from CTVp and CTVn to create the PTVp and PTVn, respectively. Volumetric modulated arc therapy (VMAT) plans were generated with two or three 360° arcs (6 MV) using the Eclipse treatment planning system (Version 15.6, Varian Medical Systems, Palo Alto, CA). Most treatments were delivered on Varian TrueBeam linear accelerators planned on a machine with a six degree‐of‐freedom (6‐DOF) couch. Seven patients were planned on Varian Clinac 21iX machines using a standard couch, all in the conventional fractionation arm. Daily CBCT was used to localize the target volumes prior to beam delivery. Patients were instructed to follow institutional bowel and bladder preparation guidelines, which require an empty rectum and a comfortably full bladder.

**FIGURE 1:**
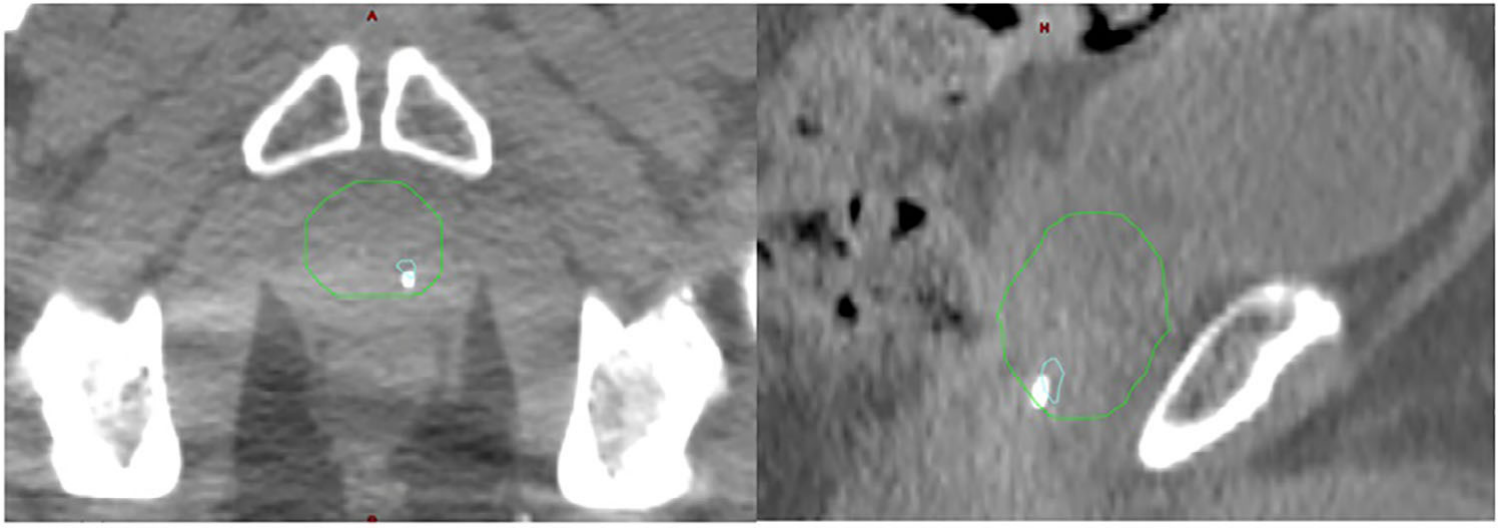
CBCT acquired during radiation treatment in the axial view (left) and sagittal view (right) from Varian's Offline Review, showing the fiducial marker residual error after the CBCT matching. Fiducial marker (blue contour); prostate contour (green).

The CBCT image matching process is as follows. Initially, radiation therapists auto‐match to bony anatomy and assess rectum and bladder filling. The prostate‐rectum interface is then matched and the fiducial marker positions assessed, while confirming that the nodal volumes remain covered by the PTVn, as much as visualized within the CBCT field of view. Therapists also confirm that the treated portion of the seminal vesicles is adequately covered by the PTVp. If any target is outside of its PTV, manual adjustment is allowed. Repeat CBCT and/or setup is performed if targets cannot be adequately matched. Because of the matching technique, the fiducial markers are not always perfectly matched. Therefore, the residual error in the prostate position can be determined by using the residual error of the fiducial marker positions. Figure 1 illustrates the residual error of a particular fiducial marker in the online match.

### 2.2 Setup error evaluation

To evaluate the setup error, we retrospectively reviewed CBCT image matching using Aria Offline Review (Version 15.6, Varian Medical Systems, Palo Alto, CA), analyzing all fractions in both treatment arms. For fractions where multiple CBCT scans were acquired (e.g., due to unacceptable rectal/bladder filling), the latest (successful) CBCT match was used for the analysis. The residual setup errors for the prostate and nodes were determined as follows. For each fraction, the average distance between each fiducial marker position in the planning CT and daily CBCT was measured for the three Cartesian coordinates, representing the residual error to the prostate match for that fraction. This was done by using the manual match function in Offline Review to match each individual marker in the CBCT to its corresponding position in the planning CT and then noting the required couch shift from the treatment position. Similarly, the residual error in the bony anatomy was determined as the distance between the online match used for treatment and a retrospective auto‐match to bone using the Hounsfield unit (HU) range for bones (200–1700 HU). This bony anatomy error was taken to represent the setup error for the nodes. A sub‐group analysis was also performed for patients treated on the standard arm, examining the impact of the 6‐DOF couch on residual errors. Similarly, we examined residual error differences between treatment arms when restricted to fractions treated on a 6‐DOF couch. In addition to calculating the residual errors, we used Offline Review to retrospectively determine the total time required for image matching as well as the number of repeat CBCT scans for each treatment arm.

### 2.3 Planning target volume estimation

The population systematic error, Σ, and random error, σ, were calculated from the residual errors as described in a report by The Royal College of Radiologists.^12^ Using the margin recipe developed by van Herk et al.^13^ the PTV margin estimation for the prostate, PTVp, and the nodes, PTVn, are defined as

(1) $$PTV_{p}=2.5\Sigma_{p}+0.7\sigma_{p}$$

(2) $$PTV_{n}=2.5\Sigma_{n}+0.7\sigma_{n}$$

Note that we are using these equations to account for residual uncertainty after daily CBCT rather than pre‐correction setup variability typically considered in the early margin recipe papers.

To account for the case of hypofractionation where the number of fractions, *N*, is relatively small, effective systematic and random errors can be approximated as^14^:

(3) $$\Sigma_{eff}^{2}=\Sigma^{2}+\sigma^{2}/N,\;\sigma_{eff}^{2}=\sigma^{2}\;\left(1-1/N\right)$$

Statistical analysis was performed using SAS version 9.4 (SAS Institute, Cary, NC, USA) using two‐sided statistical testing at the 0.05 significance level. Normality was determined using the Shapiro–Wilk test. Significance tests of centrality (mean and median) were performed using a two‐sample *t*‐test for normally distributed data; otherwise, a Wilcoxon rank sum test was used.

## 3 RESULTS

### 3.1 Residual errors and PTV margins

**FIGURE 2:**
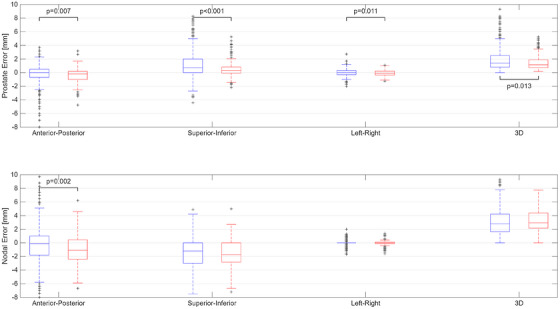
Box‐and‐whisker plots showing residual errors for (a) prostate and (b) pelvic nodes in the three cardinal directions, as well as the total magnitude (3D error). Conventional fractionation is displayed in blue (left grouping), while ultra‐hypofractionation is displayed in red (right grouping).

The residual setup errors are shown in Figure 2 for prostate and nodes in the conventional and ultra‐hypofractionated arms. In determining the bony match, we found that the entire nodal CTV was visible in 75% and 83% of CBCT scans for the conventional and ultra‐hypofractionated arms, respectively. For scans where the entire volume was not visible, the median (interquartile range [IQR]) length that was cutoff was 0.9 (0.4–1.6) cm. Statistically significant differences were observed between ultra‐hypofractionated prostate (fiducial marker) residual errors in all directions as well as the 3D magnitude (Figure 2a). For the pelvic residual errors, only the anterior‐posterior direction was statistically significantly different between the two fractionation regimens (Figure 2b). The margin calculation for prostate, PTVp, and the nodes, PTVn, is summarized in Table 1. For the PTV margin calculation in the ultra‐hypofractionation case, a correction was applied to the systematic and random errors reported in the table to account for the relatively small number of fractions, as described in Section 2. To account for setup error, the required margin for the prostate was found to be approximately 1 mm laterally and 3–4 mm in the other directions. The nodal PTV was approximately 1 mm laterally and 4–6 mm elsewhere. For the prostatic PTV, the required margins in the superior‐inferior direction was larger in the conventionally fractionated arm by 0.8 mm, while margins in the other directions differed by at most 0.2 mm. On the other hand, the nodal PTV margins for the conventionally fractionated arm were smaller in all directions by 0.3–0.5 mm.

**TABLE 1:** Systematic error, random error, and PTV margins accounting for residual setup errors for prostate (PTVp) and nodes (PTVn). Note that, to correct for the small number of fractions in the PTV margin calculation for the ultra‐hypofractionated arm, the correction described in the main text must first be applied to the uncertainties presented in this table.

	Systematic error (mm)			Random error (mm)			PTV margin size (mm)		
PTV and fractionation	AP	SI	Lat	AP	SI	Lat	AP	SI	Lat
PTVp, 25 fractions	0.85	1.15	0.22	1.02	1.32	0.37	2.8	3.8	0.8
PTVp, 5 fractions	0.72	0.92	0.27	0.95	0.87	0.37	2.7	3.0	1.0
PTVn, 25 fractions	1.65	1.32	0.18	1.83	1.64	0.33	5.4	4.4	0.7
PTVn, 5 fractions	1.62	1.42	0.30	1.82	1.53	0.30	5.7	4.9	1.0

Abbreviations: AP, anterior‐posterior; Lat, lateral; PTV, planning target volume; SI, superior‐inferior.

### 3.2 Impact of 6‐DOF couch on residual errors

For patients in the standard arm, there were 461 treatment fractions that used a 6‐DOF couch, while 187 fractions did not. For patients on the ultra‐hypofractionated arm, 139 out of 140 fractions were treated on a 6‐DOF couch. The residual errors for prostate fiducials and bony anatomy are shown in Table 2 for the standard fractionation with and without the 6‐DOF couch, as well as for conventional versus ultra‐hypofractionation when restricted to fractions treated on a 6‐DOF couch. Median 3D residual errors were 1.8 mm for the prostate when using a standard couch compared to 1.3 mm with a 6‐DOF couch (*p* < 0.001). On the other hand, median 3D residual errors were larger for the nodal target at 2.2 mm for a standard couch compared to 2.9 mm for the 6‐DOF couch (*p* = 0.007). For nodal targets, the anterior‐posterior error was greater by 0.6 mm in the ultra‐hypofractionated arm (*p* = 0.015), while errors in the other directions were not statistically significantly different. Despite the error differences for individual directions, 3D errors were not statistically significantly different for either target region.

**TABLE 2:** Residual prostate and bony anatomy errors for patients treated on the conventional fractionation arm, with or without the use of a 6‐DOF couch. Residual errors are also shown according to treatment arm, restricted to fractions treated using a 6‐DOF couch. Values are reported as median (interquartile range).

		Prostate residual errors (mm)				Bony anatomy residual errors (mm)			
Treatment	*N*	AP	SI	Lat	3D	AP	SI	Lat	3D
Conventional									
Standard couch	187	0.0 (–1.0, 0.7)	1.0 (0.0, 3.0)	0.0 (–0.3, 0.0)	1.8 (1.0, 3.5)	0.0 (–1.0, 1.0)	−1.0 (–2.0, 0.0)	0.0 (0.0, 0.0)	2.2 (1.4, 4.1)
6‐DOF couch	461	−0.1 (–0.7, 0.4)	0.6 (0.0, 1.5)	0.1 (–0.2, 0.3)	1.3 (0.7, 2.2)	−0.5 (–1.9, 1.0)	−1.5 (–3.1, –0.1)	0.0 (–0.1, 0.1)	2.9 (1.8, 4.2)
*p* value		0.35	<.001	<.001	<.001	0.04	0.008	0.53	0.007
6‐DOF couch									
Conventional fractionation	461	−0.1 (–0.7, 0.4)	0.6 (0.0, 1.5)	0.1 (–0.2, 0.3)	1.3 (0.7, 2.2)	−0.5 (–1.9, 1.0)	−1.5 (–3.1, –0.1)	0.0 (–0.1, 0.1)	2.9 (1.8, 4.2)
Ultra‐hypofractionation	139	−0.2 (–1.1, 0.2)	0.3 (–0.1, 0.8)	−0.1 (–0.4, 0.2)	1.2 (0.7, 1.9)	−1.1 (–2.4, 0.5)	−1.8 (–2.9, 0.0)	0.0 (–0.1, 0.1)	2.9 (2.1, 4.4)
*p* value		0.011	0.012	0.001	0.32	0.015	0.89	0.37	0.38

Abbreviations: 3D, three dimensional; 6‐DOF, six‐degree‐of‐freedom; Lat, lateral; *N*, number of fractions analyzed; SI, superior‐inferior.

When comparing residual errors between arms and restricting fractions treated on a 6‐DOF couch, median differences in prostate residual errors ranged from 0.1 to 0.3 mm (*p* < 0.05 in all cases).

### 3.3 Re‐imaging and image guidance timings

**FIGURE 3:**
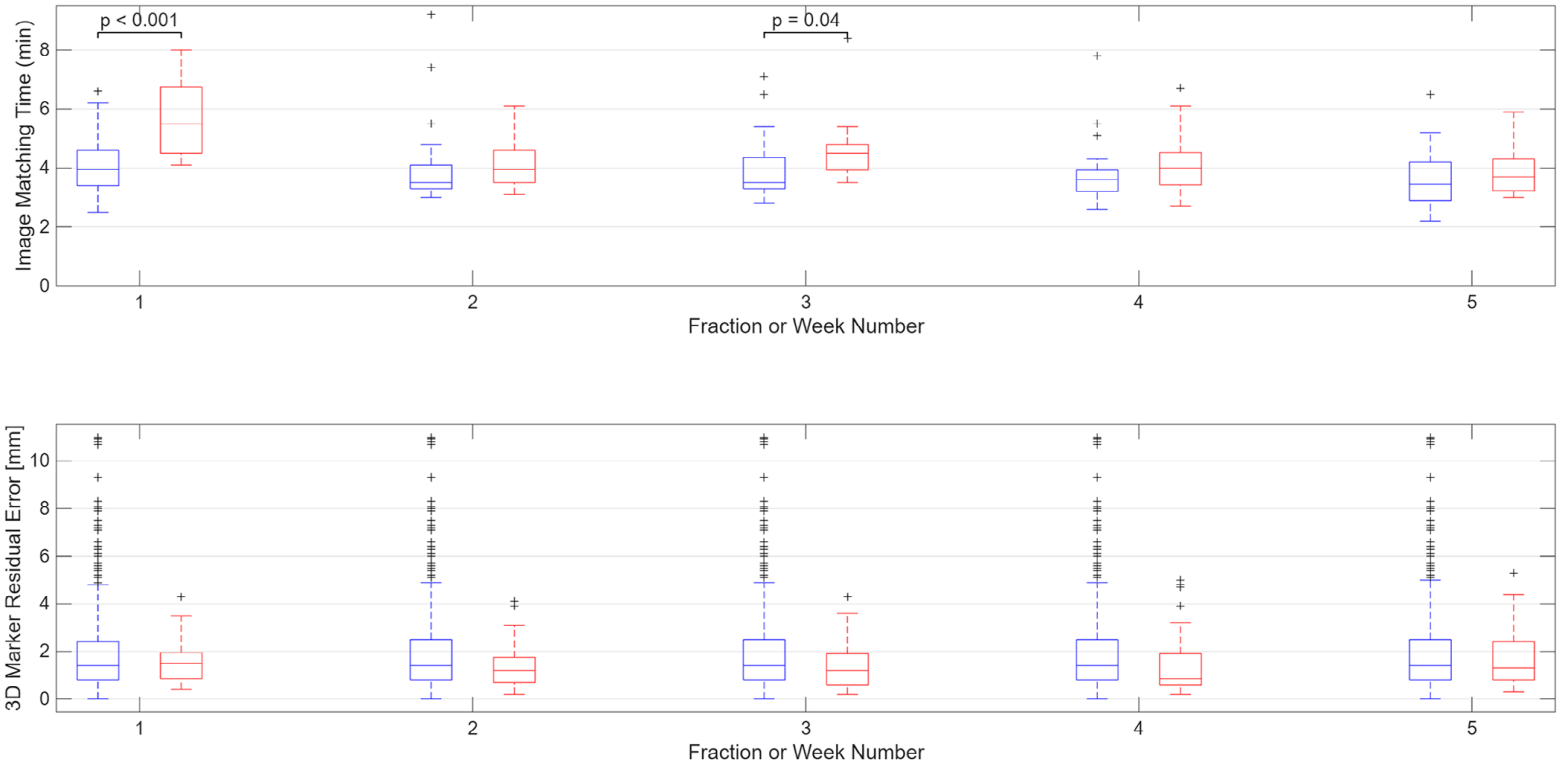
(a) Patient image matching times and (b) Mean 3D fiducial marker residual error over each individual fraction (ultra‐hypofractionated treatment) or from one selected fraction during each week (conventional treatment). Conventional fractionation is displayed in blue (left grouping), while ultra‐hypofractionation is displayed in red (right grouping).

The time required to perform the image guided radiation therapy (IGRT) matching procedure for conventional versus ultra‐hypofractionated treatments is shown in Figure 3a at five timepoints within the treatment course. These times were consistently longer for the ultra‐hypofractionated treatment, although this difference was only statistically significant at timepoints 1 and 3 (corresponding to 0% and 40% of fractions delivered). The median image matching time when there were no repeat CBCT was 4.2 and 3.5 min for the ultra‐hypofractionated and conventional treatments, respectively (*p* < 0.001). In the case of repeated patient setups (including the time where patients had to get off the treatment bed), the median time was 35.2 and 21.2 min for the ultra‐hypofractionated and conventional treatment, respectively, but this difference was not statistically significant (*p* = 0.055). Figure 3b shows the 3D error at these same timepoints. We also analyzed the number of fractions that required at least one repeat patient setup, for example, due to differences in rectal/bladder filling compared to the planning CT. There were 31 repeat setups from all 140 fractions in the ultra‐hypofractionated arm (22.1%) and 57 repeat setups from all 648 fractions in the conventionally fractionated arm (8.8%).

## 4 DISCUSSION

The HOPE trial compared conventional whole pelvis radiation therapy to ultra‐hypofractionated whole pelvis radiation therapy after HDR brachytherapy. Importantly, this trial found that ultra‐hypofractionated whole pelvis radiation therapy is as well tolerated as conventional fractionation in terms of both acute^3^ and late GI and GU toxicities.^15^ Given this positive finding and the potential for this regimen to become a standard‐of‐care treatment option, we sought here to determine whether fiducial markers (implanted at the time of prostate brachytherapy) could result in safe reduction of PTV margins.

Our results suggest that the 6‐mm nodal PTV margin size used in the trial is appropriate for both arms to account for setup errors, although this could be reduced in the lateral direction if using a non‐uniform margin. Although we did note that the nodal volume was not fully visualized for a minority of patients, the average length that was cut‐off was 1 cm compared to a typical 14 cm nodal length, and so this is not expected to significantly impact our results. In terms of the PTVp, we found that the 6‐mm PTV margin around the prostate used in HOPE trial was overly conservative for interfraction motion. Instead, a uniform PTV margin of 4 mm was found to be sufficient to account for setup error, although other sources of uncertainty need to be carefully considered. Statistically significant differences in residual errors were noted between arms, resulting in small between‐arm differences in PTV margins.

Our margin calculations for prostate and bony anatomy match are comparable to those in the work of Kaneda et al.,^16^ except they used daily MV imaging with fiducial markers along with weekly offline CT to generate their setup errors. There is good evidence that smaller PTV margins can result in reduced toxicity in the ultra‐hypofractionated radiotherapy setting. In the MIRAGE phase 3 randomized trial, for example, over 150 patients were treated with prostate stereotactic body radiation therapy (SBRT) using either CT or MR image‐guidance platforms.^17^ Patients treated with MR guidance had setup error margins reduced during planning from 4 to 2 mm, as this technology allowed for target visualization while the beam is on. With respect to the trial's primary endpoint, patients undergoing treatment with MR‐guided SBRT had a reduced incidence of grade ≥ 2+ GU toxicity when compared to the control arm (24% vs. 43%, *p* = 0.01). Likewise, the incidence of grade ≥ 2+ GI toxicity was lower in the experimental arm (0% vs. 10.5%, *p* = 0.003). In addition, there are other smaller series which further demonstrate that a reduction in PTV margins result in lower GU and GI toxicities.^18^, ^19^

The conventionally fractionated arm had a significant number of fractions treated on both a standard and 6‐DOF couch, permitting a sub‐group analysis. Interestingly, we found that the use of a 6‐DOF couch statistically significantly reduced prostate 3D residual errors at the expense of increased 3D nodal residual errors. Increased nodal errors may be the result of these uncorrected rotational errors. While not specifically analyzed here, these rotational corrections are typically less than 1° in pelvic radiation therapy.^20^ We looked at the first five patients in each arm and found that 95%, 100%, and 100% of the residual pitch, roll, and yaw angle corrections were less than 1°, respectively, for both arms. The average distance from the isocenter to the superior most slice of the PTV was roughly 8 cm, which translates to a 1.4 mm shift in the anterior‐posterior direction for a 1° rotation. Given our estimation that this happened in only 5% of the fractions, we expect that the impact of uncorrected rotations on the nodal PTV margin to be small. Statistically significant differences were seen in most directions for both the prostate and nodal volumes. When restricting the analysis to fractions treated using a 6‐DOF couch, small but statistically significant differences were seen in all directions for the prostate (0.1–0.3 mm) and in the anterior‐posterior direction for the nodes (0.6 mm), suggesting that couch‐type alone may not entirely explain the PTV margin differences that were seen between arms. It is also interesting to note that when combining these errors in 3D, differences were no longer statistically significantly different between arms, for either target region.

In terms of IGRT timings, Figure 3a illustrates that radiation therapists consistently required more time matching the ultra‐hypofractionated cases, even though the IGRT protocol was the same for both arms of the study. From Figure 3b, we observed no clear trend towards increasing or decreasing setup errors throughout the course of treatment, for either arm, although the residual errors were consistently higher for the conventional treatment. In addition, we found that there was an increased number of repeated setups in the ultra‐hypofractionated arm, with 22.1% of patients requiring a repeat CBCT compared with only 8.8% from the conventional arm. The higher dose per fraction may have led radiation therapists to spend additional time ensuring an accurate prostate match.

The results of this study should be interpreted in the context of its limitations. Importantly, our PTV margin calculation did not include potentially significant sources of uncertainty from contouring,^21^ intra‐fraction motion,^22^ and prostate deformation. Incorporation of triggered kV imaging^23^ may be required to limit intra‐fraction motion when using smaller PTVp margins, while fused MR imaging may be used to reduce contouring uncertainties. To estimate the impact of these additional sources of uncertainty on the final PTV margin, we can combine the values reported in Table 1 with numbers determined from a report by Tudor et al.^24^ For example, we find that if intra‐fraction motion is not controlled, then the PTVp margins for the ultra‐hypofractionated arm would need to be increased to 5.0/5.5/4.1 mm in the anterior‐posterior/superior‐inferior/lateral directions, respectively.

Our results are limited to institutions where daily CBCT is performed, which should be the case with any ultra‐hypofractionated regimen such as used in the HOPE trial or prostate SBRT. In addition, our findings that residual setup errors differed between treatment arms, despite following identical image matching protocol, serves to emphasize the importance of a robust IGRT program which, among other things, should include: standardized protocols and workflows that provide sufficient time for IGRT alignment; comprehensive staff training and documentation; and a comprehensive quality assurance program, including a local assessment of uncertainties affecting treatment. Variability in any of the elements will influence systematic and random errors, and so institutional PTV margins are not universally transferable. Our sub‐analysis examining the use of 6‐DOF versus a standard couch is limited by the fact that most of the 187 fractions were from only seven patients in the standard fractionation arm. Finally, while the HOPE trial was a multi‐institutional study, we were only able to analyze online CBCT matching data for patients treated at our institution which may limit the generalizability of our results.

## 5 CONCLUSION

For whole pelvis radiation therapy to the prostate and regional lymph nodes, the 6‐mm PTV margin around the nodal CTV was found to be appropriate to account for setup errors, while the margin around the prostate CTV could be reduced. The residual 3D errors for the prostate were statistically significantly smaller in the ultra‐fractionated regimen, which was potentially due to longer image matching times and/or the higher percentage of patients treated with a 6‐DOF couch in that arm. However, this came at the expense of slightly larger nodal PTV margins in the ultra‐hypofractionated arm. The results of this study highlight the need for individual centres to independently quantify their own uncertainties, as measurable differences were observed between treatment arms despite identical image guidance protocols.

## AUTHOR CONTRIBUTIONS

Bryan Schaly is the primary author of the manuscript, developed the project plan, data acquisition, and analysis. Lucas C. Mendez provided clinical trial data, developed project plan, and reviewed manuscript. Maha Khawaja contributed to data acquisition and analysis and reviewed the manuscript. Alec Black contributed additional data acquisition and analysis and reviewed the revised manuscript. Andrew Warner performed statistical analysis and reviewed the manuscript. Douglas A. Hoover developed the project plan, performed the data analysis, and reviewed the manuscript.

## ETHICS STATEMENT

The HOPE clinical trial was approved by Western University Ethics REB #114388.

## CONFLICT OF INTEREST STATEMENT

The authors have no relevant conflict of interest to disclose.

## Data Availability

The trial protocol did not include a data sharing plan and therefore data from the trial will not be shared publicly, as sharing was not included in the ethics approvals.
